# 9-[(*E*)-2-(4-Chloro­phen­yl)ethen­yl]-3,3,6,6-tetra­methyl-2,3,4,5,6,7,8,9-octa­hydro-1*H*-xanthene-1,8-dione

**DOI:** 10.1107/S1600536813025026

**Published:** 2013-09-18

**Authors:** Byung-Yong Yu, Jae Kyun Lee, Yong Seo Cho, Sun-Joon Min, Jae Chun Woo

**Affiliations:** a Advanced Analysis Center, Korea Institute of Science & Technology, Hwarangro 14-gil, Seongbuk-gu, Seoul, 136-791, South Korea; b Center for Neuro-Medicine, Korea Institute of Science & Technology, Hwarangro 14-gil, Seongbuk-gu, Seoul, 136-791, South Korea; c Drug Discovery Platform Technology Team, Korea Research Institute, of Chemical Technology, PO Box 107, Yuseong, Daejeon 305-600, South Korea

## Abstract

In the title compound, C_25_H_27_ClO_3_, each of the cyclo­hexenone rings adopts an envelope conformation, whereas the six-membered pyran ring adopts a flattened boat conformation, with the O and methine C atoms deviating from the plane of the other four atoms. The C=C double bond is in the *trans* conformation. In the crystal, weak C—H⋯O hydrogen bonds link the mol­ecules into chains running parallel to the *b* axis.

## Related literature

For the synthesis and the crystal structures of xanthene derivatives studied recently by our group, see: Cha *et al.* (2013 ▶); Lee *et al.* (2012 ▶, 2013 ▶).
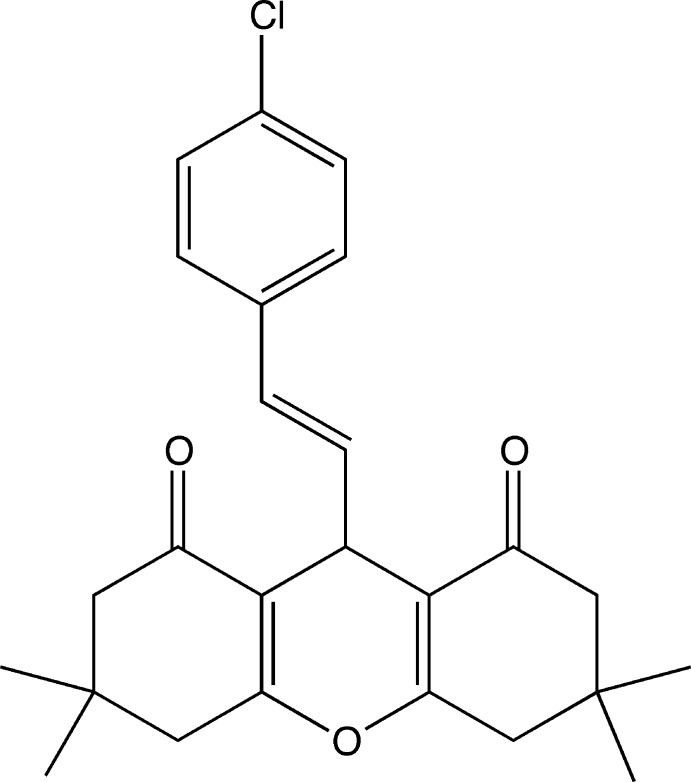


## Experimental

### 

#### Crystal data

C_25_H_27_ClO_3_
*M* *_r_* = 410.94
Monoclinic, 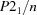

*a* = 5.9686 (4) Å
*b* = 18.7567 (13) Å
*c* = 20.1089 (13) Å
β = 100.9322 (18)°
*V* = 2210.4 (3) Å^3^
*Z* = 4
Mo *K*α radiation
μ = 0.20 mm^−1^
*T* = 296 K
0.40 × 0.10 × 0.10 mm

#### Data collection

Rigaku R-AXIS RAPID diffractometer
Absorption correction: multi-scan (*ABSCOR*, Rigaku, 1995 ▶) *T*_min_ = 0.629, *T*_max_ = 0.981
21185 measured reflections
5042 independent reflections
2842 reflections with *F* ^2^ > 2.0σ(*F* ^2^)
*R*_int_ = 0.042

#### Refinement

*R*[*F* ^2^ > 2σ(*F* ^2^)] = 0.057
*wR*(*F* ^2^) = 0.194
*S* = 1.11
5042 reflections
274 parameters
H atoms treated by a mixture of independent and constrained refinement
Δρ_max_ = 0.49 e Å^−3^
Δρ_min_ = −0.37 e Å^−3^

### 

Data collection: *RAPID-AUTO* (Rigaku, 2006 ▶); cell refinement: *RAPID-AUTO*; data reduction: *RAPID-AUTO*; program(s) used to solve structure: *Il Milione* (Burla *et al.*, 2007 ▶); program(s) used to refine structure: *SHELXL97* (Sheldrick, 2008 ▶); molecular graphics: *CrystalStructure* (Rigaku, 2010 ▶); software used to prepare material for publication: *CrystalStructure*.

## Supplementary Material

Crystal structure: contains datablock(s) General, I. DOI: 10.1107/S1600536813025026/ff2119sup1.cif

Structure factors: contains datablock(s) I. DOI: 10.1107/S1600536813025026/ff2119Isup2.hkl

Supplementary material file. DOI: 10.1107/S1600536813025026/ff2119Isup3.cml

Additional supplementary materials:  crystallographic information; 3D view; checkCIF report

## Figures and Tables

**Table 1:** Hydrogen-bond geometry (Å, °)

*D*—H⋯*A*	*D*—H	H⋯*A*	*D*⋯*A*	*D*—H⋯*A*
C23—H23*B*⋯O2^i^	0.96	2.53	3.461 (4)	163

Symmetry code: (i) 
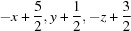
.

**Fig. 1.**
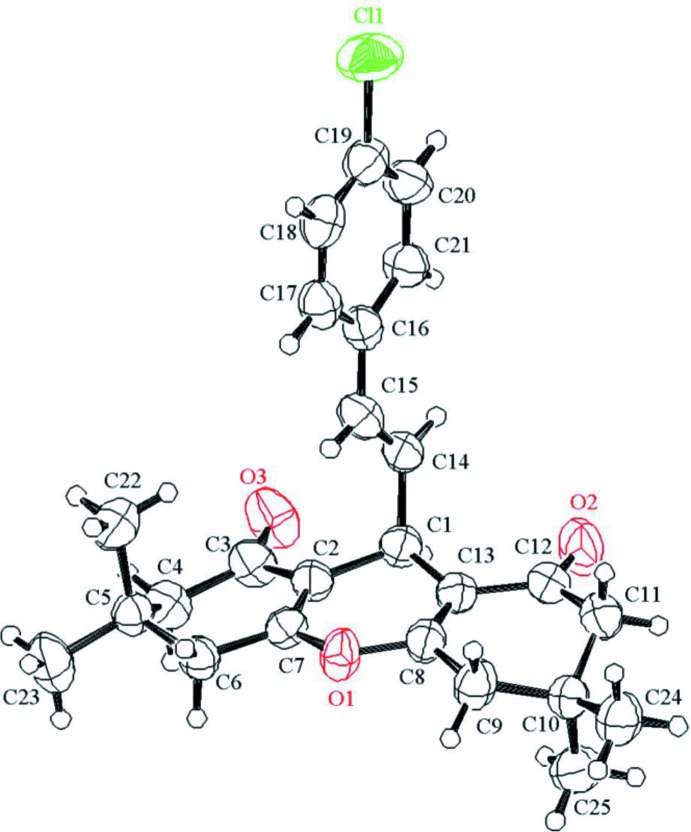
The molecular structure of (I) showing the atomic numbering and 50% probability displacement ellipsoids.

## supplementary crystallographic information

### 1. Comment

As part of our ongoing study of the substituent effect on the solid state structures of xanthene derivatives (Cha *et al.*, 2013; Lee *et al.*, 2012, 2013). We present here the crystal structure of the title compound (I) (Fig. 1).

The starting material, (*E*)-2.2-(3-(4-chlorophenyl)prop-2-ene-1,1-diyl) bis(3-hydroxy-5,5-δimethylcyclohex-2-enone) was prepared according to the reported method (Cha *et al.*, 2013). In (I) (Fig. 1), the bond lengths and angles are normal and correspond to those observed in related structures (Lee *et al.*, 2013).

The torsion angle C2—C1—C14—C15 between the benzene ring (C16 - C21) system and the pyran ring (C1—C2—C7—O3—C8—C13) is 41.74 (2)°. The double bond [C14=C15] is in the *trans* conformation. All two cyclohexenone rings in (Fig.1) display envelope conformation, whereas the pyran ring adopts a boat conformation.

In the crystal, weak intermolecular C—H···O hydrogen bonds link molecules into chains running parallel to the *b*-axis.

### 2. Experimental

To solution of (*E*)-2.2-(3-(4-Chlorophenyl)prop-2-ene-1,1-diyl)bis (3-hydroxy-5,5-dimethylcyclohex-2-enone) (1.25 mmol) was added methanol and catalytic amounts of sulfuric acid in under nitrogen atmosphere. After stirring for 4 h, the progress of reaction was monitored by TLC. The solvent was evaporated and the remaining residue dissolved in ethyl acetate. The mixture was neutralized with saturated sodium bicarbonate and the solution was extracted with ethyl acetate. The resulting solid residue was purified by recrystallization from ethanol and methylene chloride (1:7 v/v) to afford (yield 95%) colorless block type crystals suitable for X-ray analysis.

### 3. Refinement

All hydrogen atoms were positioned geometrically and refined using a riding model with C—H = 0.86–0.98 Å and *U*iso(H) = 1.2 or 1.5 *U*eq(C).

### Crystal data

**Table:**

C_25_H_27_ClO_3_	*F*(000) = 872.00
*M**_r_* = 410.94	*D*_x_ = 1.235 Mg m^−^^3^
Monoclinic, *P*2_1_/*n*	Mo *K*α radiation, λ = 0.71075 Å
Hall symbol: -P 2yn	Cell parameters from 12401 reflections
*a* = 5.9686 (4) Å	θ = 3.0–27.5°
*b* = 18.7567 (13) Å	µ = 0.20 mm^−^^1^
*c* = 20.1089 (13) Å	*T* = 296 K
β = 100.9322 (18)°	Block, colorless
*V* = 2210.4 (3) Å^3^	0.40 × 0.10 × 0.10 mm
*Z* = 4	

### Data collection

**Table:**

Rigaku R-AXIS RAPID diffractometer	2842 reflections with *F*^2^ > 2.0σ(*F*^2^)
Detector resolution: 10.000 pixels mm^-1^	*R*_int_ = 0.042
ω scans	θ_max_ = 27.5°
Absorption correction: multi-scan (*ABSCOR*, Rigaku, 1995)	*h* = −7→7
*T*_min_ = 0.629, *T*_max_ = 0.981	*k* = −24→24
21185 measured reflections	*l* = −26→26
5042 independent reflections	

### Refinement

**Table:**

Refinement on *F*^2^	Secondary atom site location: difference Fourier map
*R*[*F*^2^ > 2σ(*F*^2^)] = 0.057	Hydrogen site location: inferred from neighbouring sites
*wR*(*F*^2^) = 0.194	H atoms treated by a mixture of independent and constrained refinement
*S* = 1.11	*w* = 1/[σ^2^(*F*_o_^2^) + (0.0909*P*)^2^ + 0.3774*P*] where *P* = (*F*_o_^2^ + 2*F*_c_^2^)/3
5042 reflections	(Δ/σ)_max_ < 0.001
274 parameters	Δρ_max_ = 0.49 e Å^−^^3^
0 restraints	Δρ_min_ = −0.37 e Å^−^^3^
Primary atom site location: structure-invariant direct methods	

### Special details

**Table:**

Geometry. All e.s.d.'s (except the e.s.d. in the dihedral angle between two l.s. planes) are estimated using the full covariance matrix. The cell e.s.d.'s are taken into account individually in the estimation of e.s.d.'s in distances, angles and torsion angles; correlations between e.s.d.'s in cell parameters are only used when they are defined by crystal symmetry. An approximate (isotropic) treatment of cell e.s.d.'s is used for estimating e.s.d.'s involving l.s. planes.
Refinement. Refinement was performed using all reflections. The weighted *R*-factor (*wR*) and goodness of fit (*S*) are based on *F*^2^. *R*-factor (gt) are based on *F*. The threshold expression of *F*^2^ > 2.0 σ(*F*^2^) is used only for calculating *R*-factor (gt).

### Fractional atomic coordinates and isotropic or equivalent isotropic displacement parameters (Å^2^)

**Table:**

	*x*	*y*	*z*	*U*_iso_*/*U*_eq_	
Cl1	0.69966 (17)	0.07641 (5)	1.05177 (4)	0.0940 (4)	
O1	0.7005 (3)	0.30837 (9)	0.63125 (9)	0.0555 (5)	
O2	1.1309 (4)	0.09617 (11)	0.64406 (11)	0.0807 (7)	
O3	1.4226 (3)	0.33059 (11)	0.77323 (11)	0.0772 (7)	
C1	1.0997 (4)	0.23354 (12)	0.70199 (12)	0.0475 (6)	
C2	1.0643 (4)	0.31308 (12)	0.70432 (11)	0.0445 (6)	
C3	1.2468 (4)	0.35734 (14)	0.74179 (14)	0.0553 (7)	
C4	1.2148 (5)	0.43693 (14)	0.73819 (14)	0.0574 (7)	
C5	0.9668 (4)	0.46069 (12)	0.73584 (12)	0.0465 (6)	
C6	0.8129 (4)	0.42116 (12)	0.67791 (12)	0.0476 (6)	
C7	0.8714 (4)	0.34484 (12)	0.67331 (11)	0.0433 (5)	
C8	0.7416 (4)	0.23900 (13)	0.61490 (12)	0.0502 (6)	
C9	0.5527 (5)	0.21123 (15)	0.56166 (13)	0.0594 (7)	
C10	0.6217 (5)	0.14590 (12)	0.52433 (12)	0.0497 (6)	
C11	0.7561 (5)	0.09527 (13)	0.57644 (14)	0.0623 (7)	
C12	0.9542 (5)	0.12910 (13)	0.62313 (13)	0.0560 (7)	
C13	0.9267 (4)	0.20277 (13)	0.64482 (11)	0.0478 (6)	
C14	1.0738 (5)	0.19832 (13)	0.76848 (12)	0.0502 (6)	
C15	0.9167 (5)	0.21453 (14)	0.80290 (14)	0.0540 (7)	
C16	0.8714 (4)	0.18080 (12)	0.86494 (12)	0.0477 (6)	
C17	0.6773 (5)	0.20050 (14)	0.88966 (13)	0.0563 (7)	
C18	0.6241 (5)	0.16827 (14)	0.94691 (14)	0.0607 (7)	
C19	0.7662 (5)	0.11714 (15)	0.98006 (13)	0.0587 (7)	
C20	0.9630 (5)	0.09732 (14)	0.95799 (14)	0.0605 (7)	
C21	1.0122 (4)	0.12912 (13)	0.90070 (13)	0.0539 (6)	
C22	0.9015 (5)	0.44069 (16)	0.80400 (14)	0.0677 (8)	
C23	0.9392 (6)	0.54004 (15)	0.72602 (18)	0.0790 (9)	
C24	0.4088 (5)	0.10911 (16)	0.48605 (15)	0.0692 (8)	
C25	0.7708 (5)	0.16842 (16)	0.47402 (14)	0.0694 (8)	
H1	1.2533	0.2240	0.6936	0.0570*	
H4A	1.2650	0.4546	0.6981	0.0689*	
H4B	1.3110	0.4584	0.7774	0.0689*	
H6A	0.6560	0.4249	0.6840	0.0572*	
H6B	0.8234	0.4443	0.6355	0.0572*	
H9A	0.5029	0.2488	0.5290	0.0713*	
H9B	0.4244	0.1986	0.5826	0.0713*	
H11A	0.6532	0.0753	0.6035	0.0747*	
H11B	0.8129	0.0562	0.5527	0.0747*	
H17	0.5819	0.2358	0.8675	0.0676*	
H18	0.4930	0.1814	0.9625	0.0728*	
H20	1.0603	0.0632	0.9814	0.0726*	
H21	1.1437	0.1156	0.8855	0.0647*	
H22A	0.7482	0.4560	0.8042	0.0813*	
H22B	1.0041	0.4637	0.8402	0.0813*	
H22C	0.9116	0.3899	0.8100	0.0813*	
H23A	0.9751	0.5531	0.6831	0.0948*	
H23B	1.0404	0.5643	0.7616	0.0948*	
H23C	0.7845	0.5532	0.7271	0.0948*	
H24A	0.4521	0.0680	0.4631	0.0831*	
H24B	0.3253	0.1415	0.4535	0.0831*	
H24C	0.3146	0.0946	0.5174	0.0831*	
H25A	0.9061	0.1914	0.4979	0.0833*	
H25B	0.6873	0.2009	0.4416	0.0833*	
H25C	0.8126	0.1271	0.4510	0.0833*	
H14	1.170 (5)	0.1623 (16)	0.7859 (15)	0.073 (9)*	
H15	0.820 (5)	0.2468 (16)	0.7866 (15)	0.069 (9)*	

### Atomic displacement parameters (Å^2^)

**Table:**

	*U*^11^	*U*^22^	*U*^33^	*U*^12^	*U*^13^	*U*^23^
Cl1	0.1078 (7)	0.1136 (8)	0.0698 (6)	0.0045 (6)	0.0402 (5)	0.0178 (5)
O1	0.0479 (9)	0.0512 (11)	0.0609 (11)	0.0086 (8)	−0.0067 (8)	−0.0155 (8)
O2	0.0945 (16)	0.0552 (12)	0.0814 (15)	0.0261 (11)	−0.0114 (12)	−0.0069 (10)
O3	0.0510 (11)	0.0622 (13)	0.1048 (17)	0.0042 (9)	−0.0200 (11)	0.0069 (11)
C1	0.0466 (13)	0.0483 (14)	0.0452 (13)	0.0060 (11)	0.0026 (10)	−0.0004 (10)
C2	0.0430 (12)	0.0453 (13)	0.0438 (12)	0.0024 (10)	0.0042 (10)	0.0012 (10)
C3	0.0445 (13)	0.0549 (15)	0.0628 (16)	0.0012 (12)	0.0011 (12)	0.0043 (12)
C4	0.0513 (14)	0.0562 (16)	0.0638 (16)	−0.0055 (12)	0.0085 (13)	0.0025 (13)
C5	0.0452 (12)	0.0452 (13)	0.0476 (13)	0.0006 (10)	0.0046 (10)	−0.0022 (10)
C6	0.0459 (13)	0.0447 (13)	0.0508 (13)	0.0036 (10)	0.0053 (11)	−0.0010 (10)
C7	0.0391 (11)	0.0488 (13)	0.0411 (12)	0.0004 (10)	0.0048 (10)	−0.0012 (10)
C8	0.0560 (14)	0.0462 (14)	0.0476 (13)	0.0030 (11)	0.0076 (11)	−0.0064 (11)
C9	0.0561 (15)	0.0594 (16)	0.0573 (15)	0.0022 (13)	−0.0029 (13)	−0.0121 (13)
C10	0.0570 (14)	0.0444 (13)	0.0465 (13)	−0.0044 (11)	0.0069 (11)	−0.0026 (11)
C11	0.0803 (19)	0.0438 (14)	0.0594 (16)	−0.0041 (13)	0.0049 (15)	−0.0004 (12)
C12	0.0721 (17)	0.0468 (14)	0.0474 (14)	0.0072 (13)	0.0065 (13)	0.0039 (11)
C13	0.0533 (14)	0.0469 (13)	0.0419 (12)	0.0055 (11)	0.0057 (11)	0.0002 (10)
C14	0.0568 (14)	0.0438 (14)	0.0465 (13)	0.0101 (12)	0.0008 (12)	0.0034 (11)
C15	0.0509 (14)	0.0466 (15)	0.0622 (16)	0.0073 (12)	0.0050 (13)	0.0095 (12)
C16	0.0491 (13)	0.0408 (13)	0.0517 (13)	0.0001 (10)	0.0061 (11)	−0.0020 (10)
C17	0.0530 (14)	0.0498 (14)	0.0639 (16)	0.0049 (12)	0.0054 (13)	−0.0021 (12)
C18	0.0549 (15)	0.0623 (17)	0.0669 (17)	0.0020 (13)	0.0167 (14)	−0.0144 (14)
C19	0.0643 (16)	0.0610 (16)	0.0529 (15)	−0.0059 (13)	0.0165 (13)	−0.0062 (13)
C20	0.0640 (16)	0.0597 (17)	0.0574 (16)	0.0091 (13)	0.0104 (14)	0.0086 (13)
C21	0.0518 (14)	0.0550 (15)	0.0566 (15)	0.0062 (12)	0.0142 (12)	0.0048 (12)
C22	0.0724 (18)	0.0711 (19)	0.0613 (17)	−0.0038 (15)	0.0167 (15)	−0.0112 (14)
C23	0.081 (2)	0.0485 (17)	0.097 (3)	−0.0016 (15)	−0.0104 (18)	−0.0047 (16)
C24	0.0748 (19)	0.0643 (18)	0.0657 (18)	−0.0121 (15)	0.0059 (15)	−0.0137 (14)
C25	0.0766 (19)	0.075 (2)	0.0565 (16)	−0.0090 (16)	0.0124 (15)	0.0023 (14)

### Geometric parameters (Å, º)

**Table:**

Cl1—C19	1.743 (3)	C19—C20	1.383 (5)
O1—C7	1.377 (3)	C20—C21	1.377 (4)
O1—C8	1.375 (3)	C1—H1	0.980
O2—C12	1.226 (4)	C4—H4A	0.970
O3—C3	1.225 (3)	C4—H4B	0.970
C1—C2	1.509 (4)	C6—H6A	0.970
C1—C13	1.507 (3)	C6—H6B	0.970
C1—C14	1.525 (4)	C9—H9A	0.970
C2—C3	1.460 (4)	C9—H9B	0.970
C2—C7	1.341 (3)	C11—H11A	0.970
C3—C4	1.505 (4)	C11—H11B	0.970
C4—C5	1.538 (4)	C14—H14	0.91 (3)
C5—C6	1.531 (3)	C15—H15	0.86 (3)
C5—C22	1.541 (4)	C17—H17	0.930
C5—C23	1.506 (4)	C18—H18	0.930
C6—C7	1.481 (4)	C20—H20	0.930
C8—C9	1.494 (4)	C21—H21	0.930
C8—C13	1.339 (4)	C22—H22A	0.960
C9—C10	1.534 (4)	C22—H22B	0.960
C10—C11	1.525 (4)	C22—H22C	0.960
C10—C24	1.521 (4)	C23—H23A	0.960
C10—C25	1.528 (5)	C23—H23B	0.960
C11—C12	1.504 (4)	C23—H23C	0.960
C12—C13	1.468 (4)	C24—H24A	0.960
C14—C15	1.303 (4)	C24—H24B	0.960
C15—C16	1.469 (4)	C24—H24C	0.960
C16—C17	1.395 (4)	C25—H25A	0.960
C16—C21	1.390 (4)	C25—H25B	0.960
C17—C18	1.389 (4)	C25—H25C	0.960
C18—C19	1.367 (4)		
			
O1···C1	2.896 (3)	O3···H17^iii^	2.6407
O2···C1	2.848 (4)	O3···H22A^iii^	3.0396
O2···C8	3.522 (4)	O3···H22C^iii^	3.0799
O2···C14	3.220 (4)	O3···H15^iii^	2.82 (3)
O3···C1	2.834 (3)	C1···H9B^iii^	3.4232
O3···C7	3.525 (3)	C3···H6A^iii^	3.1619
O3···C14	3.228 (4)	C3···H22A^iii^	3.5402
C2···C5	2.923 (4)	C4···H6A^iii^	3.0432
C2···C8	2.750 (3)	C4···H22A^iii^	3.2287
C2···C15	2.964 (4)	C6···H4A^i^	3.4288
C2···C22	3.381 (4)	C8···H1^i^	3.5812
C3···C6	2.920 (4)	C9···H1^i^	3.4761
C3···C14	3.234 (4)	C11···H22A^iv^	3.5505
C3···C22	3.039 (5)	C11···H22B^iv^	3.5075
C4···C7	2.806 (4)	C11···H24A^x^	3.3429
C7···C13	2.759 (4)	C12···H9B^iii^	3.3330
C7···C14	3.435 (4)	C12···H24C^iii^	3.3615
C7···C15	3.546 (4)	C13···H9B^iii^	3.4364
C7···C22	3.161 (4)	C14···H17^iii^	3.3706
C8···C11	2.811 (4)	C14···H23B^ii^	3.5372
C8···C14	3.425 (4)	C14···H23C^iv^	3.4729
C8···C25	3.162 (4)	C15···H23C^iv^	3.2686
C9···C12	2.919 (4)	C15···H25B^viii^	3.3457
C10···C13	2.941 (3)	C16···H9A^viii^	3.5021
C12···C14	3.154 (4)	C16···H23A^iv^	3.1917
C12···C25	3.080 (4)	C16···H23C^iv^	3.0596
C13···C15	3.198 (4)	C16···H25B^viii^	3.1228
C13···C25	3.445 (4)	C17···H9A^viii^	3.2356
C14···C21	3.043 (4)	C17···H21^i^	3.5474
C16···C19	2.780 (4)	C17···H23A^iv^	3.1784
C17···C20	2.767 (4)	C17···H24B^viii^	3.2834
C18···C21	2.754 (4)	C17···H25A^vii^	3.5780
O1···O3^i^	3.588 (3)	C17···H25B^viii^	3.5459
O2···C23^ii^	3.461 (4)	C17···H14^i^	3.41 (3)
O3···O1^iii^	3.588 (3)	C18···H9A^viii^	2.9736
O3···C17^iii^	3.521 (4)	C18···H21^i^	3.0630
O3···C22^iii^	3.485 (4)	C18···H23A^iv^	3.3557
C16···C23^iv^	3.531 (4)	C18···H25A^vii^	3.1900
C17···O3^i^	3.521 (4)	C18···H25B^vii^	3.5671
C22···O3^i^	3.485 (4)	C19···H9A^viii^	2.9604
C23···O2^v^	3.461 (4)	C19···H20^ix^	3.5788
C23···C16^vi^	3.531 (4)	C19···H23A^iv^	3.5336
Cl1···H18	2.7888	C20···H9A^viii^	3.2097
Cl1···H20	2.8027	C20···H18^iii^	3.5198
O1···H6A	2.4660	C20···H20^ix^	3.2609
O1···H6B	2.6503	C20···H23A^iv^	3.5721
O1···H9A	2.4389	C21···H9A^viii^	3.4585
O1···H9B	2.7017	C21···H18^iii^	3.0641
O1···H15	3.28 (3)	C21···H23A^iv^	3.3869
O2···H1	2.6456	C21···H23C^iv^	3.1681
O2···H11A	2.8399	C21···H25B^viii^	3.4071
O2···H11B	2.4933	C22···H4B^i^	3.4792
O2···H25A	3.4838	C22···H11A^vi^	3.1893
O2···H14	3.08 (3)	C22···H24A^viii^	3.1594
O3···H1	2.6400	C22···H24B^viii^	3.4848
O3···H4A	2.8353	C23···H14^v^	3.31 (3)
O3···H4B	2.4940	C24···H11B^x^	3.4035
O3···H22C	3.4562	C24···H22B^xi^	3.3790
O3···H14	3.53 (3)	C24···H22C^xi^	3.5428
C1···H15	2.61 (4)	C24···H24A^x^	3.5277
C2···H4A	2.9246	C24···H25A^i^	3.4227
C2···H4B	3.3067	C24···H25C^i^	3.5105
C2···H6A	3.1828	C25···H17^xii^	3.5745
C2···H6B	3.0478	C25···H18^xii^	3.1411
C2···H22C	2.8571	C25···H24B^iii^	3.4493
C2···H14	3.27 (3)	C25···H24C^iii^	3.4838
C2···H15	2.71 (3)	H1···O1^iii^	3.5308
C3···H1	2.6851	H1···C8^iii^	3.5812
C3···H6B	3.4013	H1···C9^iii^	3.4761
C3···H22B	3.3286	H1···H9B^iii^	2.6676
C3···H22C	2.7018	H1···H23B^ii^	3.2982
C3···H15	3.53 (3)	H1···H15^iii^	3.5668
C4···H6A	3.3141	H4A···Cl1^xii^	2.9523
C4···H6B	2.8128	H4A···C6^iii^	3.4288
C4···H22A	3.3239	H4A···H6A^iii^	2.4673
C4···H22B	2.6496	H4A···H21^v^	3.5481
C4···H22C	2.6700	H4A···H22A^iii^	3.2460
C4···H23A	2.7246	H4A···H23C^iii^	3.5624
C4···H23B	2.6835	H4B···O2^v^	3.0143
C4···H23C	3.3444	H4B···C22^iii^	3.4792
C6···H4A	2.7247	H4B···H6A^iii^	3.1024
C6···H4B	3.3272	H4B···H22A^iii^	2.5631
C6···H22A	2.7202	H6A···Cl1^xi^	3.4267
C6···H22B	3.3433	H6A···O3^i^	3.0391
C6···H22C	2.6749	H6A···C3^i^	3.1619
C6···H23A	2.6530	H6A···C4^i^	3.0432
C6···H23B	3.3202	H6A···H4A^i^	2.4673
C6···H23C	2.6843	H6A···H4B^i^	3.1024
C7···H1	3.1850	H6B···Cl1^xii^	3.0761
C7···H4A	3.0922	H6B···H20^xi^	3.2003
C7···H22A	3.5430	H9A···C16^xi^	3.5021
C7···H22C	2.8428	H9A···C17^xi^	3.2356
C7···H15	2.99 (3)	H9A···C18^xi^	2.9736
C8···H1	3.1742	H9A···C19^xi^	2.9604
C8···H11A	3.1166	H9A···C20^xi^	3.2097
C8···H25A	2.8589	H9A···C21^xi^	3.4585
C8···H25B	3.5123	H9A···H18^xi^	3.3500
C8···H15	3.40 (3)	H9B···O2^i^	3.0186
C9···H11A	2.7160	H9B···C1^i^	3.4232
C9···H11B	3.3174	H9B···C12^i^	3.3330
C9···H24A	3.3259	H9B···C13^i^	3.4364
C9···H24B	2.6757	H9B···H1^i^	2.6676
C9···H24C	2.6673	H9B···H25A^i^	3.2393
C9···H25A	2.6941	H11A···O2^i^	3.3915
C9···H25B	2.6929	H11A···C22^iv^	3.1893
C9···H25C	3.3406	H11A···H22A^iv^	2.8952
C11···H9A	3.3070	H11A···H22B^iv^	2.6364
C11···H9B	2.7901	H11A···H23B^iv^	3.1498
C11···H24A	2.6805	H11A···H23C^iv^	3.3821
C11···H24B	3.3260	H11A···H24A^x^	3.0146
C11···H24C	2.6775	H11B···C24^x^	3.4035
C11···H25A	2.6618	H11B···H22A^iv^	3.5133
C11···H25B	3.3206	H11B···H22B^iv^	3.5745
C11···H25C	2.6749	H11B···H24A^x^	2.7995
C12···H1	2.7208	H11B···H24C^iii^	3.2856
C12···H9B	3.3743	H11B···H24C^x^	3.1883
C12···H25A	2.7419	H17···O3^i^	2.6407
C12···H25C	3.4042	H17···C14^i^	3.3706
C12···H14	3.34 (3)	H17···C25^vii^	3.5745
C13···H9A	3.2143	H17···H21^i^	3.5230
C13···H9B	3.0225	H17···H23A^iv^	3.5712
C13···H11A	2.9252	H17···H24B^viii^	3.0741
C13···H11B	3.3124	H17···H25A^vii^	3.2996
C13···H25A	2.9417	H17···H25B^vii^	3.2416
C13···H14	3.03 (3)	H17···H14^i^	3.0182
C13···H15	3.15 (3)	H18···C20^i^	3.5198
C14···H21	2.7825	H18···C21^i^	3.0641
C15···H1	3.2524	H18···C25^vii^	3.1411
C15···H17	2.6117	H18···H9A^viii^	3.3500
C15···H21	2.6798	H18···H20^i^	3.4782
C15···H22C	3.2936	H18···H21^i^	2.6544
C16···H18	3.2612	H18···H25A^vii^	2.5705
C16···H20	3.2588	H18···H25B^vii^	2.8446
C16···H14	2.62 (4)	H20···Cl1^ix^	3.1167
C17···H21	3.2218	H20···C19^ix^	3.5788
C17···H15	2.54 (3)	H20···C20^ix^	3.2609
C18···H20	3.2342	H20···H6B^viii^	3.2003
C19···H17	3.2162	H20···H18^iii^	3.4782
C19···H21	3.2126	H20···H20^ix^	2.6271
C20···H18	3.2345	H21···C17^iii^	3.5474
C21···H17	3.2241	H21···C18^iii^	3.0630
C21···H14	2.72 (4)	H21···H4A^ii^	3.5481
C21···H15	3.23 (3)	H21···H17^iii^	3.5230
C22···H4A	3.3256	H21···H18^iii^	2.6544
C22···H4B	2.6204	H21···H23A^ii^	3.1049
C22···H6A	2.5916	H21···H23C^iv^	3.2903
C22···H6B	3.3326	H22A···O3^i^	3.0396
C22···H23A	3.3103	H22A···C3^i^	3.5402
C22···H23B	2.6569	H22A···C4^i^	3.2287
C22···H23C	2.6320	H22A···C11^vi^	3.5505
C23···H4A	2.6603	H22A···H4A^i^	3.2460
C23···H4B	2.7322	H22A···H4B^i^	2.5631
C23···H6A	2.7737	H22A···H11A^vi^	2.8952
C23···H6B	2.5571	H22A···H11B^vi^	3.5133
C23···H22A	2.6338	H22A···H24A^viii^	3.2266
C23···H22B	2.6719	H22A···H24B^viii^	3.4712
C23···H22C	3.3035	H22B···O2^v^	3.2809
C24···H9A	2.7818	H22B···C11^vi^	3.5075
C24···H9B	2.5546	H22B···C24^viii^	3.3790
C24···H11A	2.6079	H22B···H11A^vi^	2.6364
C24···H11B	2.7150	H22B···H11B^vi^	3.5745
C24···H25A	3.3138	H22B···H24A^viii^	2.6154
C24···H25B	2.6629	H22B···H24B^viii^	3.3381
C24···H25C	2.6575	H22B···H25C^viii^	3.1132
C25···H9A	2.5925	H22C···O3^i^	3.0799
C25···H9B	3.3273	H22C···C24^viii^	3.5428
C25···H11A	3.3190	H22C···H24A^viii^	3.1416
C25···H11B	2.6161	H22C···H24B^viii^	3.0828
C25···H24A	2.6568	H22C···H25B^viii^	3.3101
C25···H24B	2.6603	H22C···H25C^viii^	3.3602
C25···H24C	3.3152	H23A···C16^vi^	3.1917
H1···H14	2.3218	H23A···C17^vi^	3.1784
H1···H15	3.4931	H23A···C18^vi^	3.3557
H4A···H6B	2.7040	H23A···C19^vi^	3.5336
H4A···H22B	3.5081	H23A···C20^vi^	3.5721
H4A···H22C	3.5755	H23A···C21^vi^	3.3869
H4A···H23A	2.5103	H23A···H17^vi^	3.5712
H4A···H23B	2.8831	H23A···H21^v^	3.1049
H4A···H23C	3.5521	H23A···H14^v^	2.9290
H4B···H22A	3.5021	H23B···O2^v^	2.5279
H4B···H22B	2.4168	H23B···C14^v^	3.5372
H4B···H22C	2.8907	H23B···H1^v^	3.2982
H4B···H23A	3.0541	H23B···H11A^vi^	3.1498
H4B···H23B	2.5417	H23B···H14^v^	2.8139
H4B···H23C	3.5853	H23C···C14^vi^	3.4729
H6A···H22A	2.4458	H23C···C15^vi^	3.2686
H6A···H22B	3.4981	H23C···C16^vi^	3.0596
H6A···H22C	2.7774	H23C···C21^vi^	3.1681
H6A···H23A	3.0704	H23C···H4A^i^	3.5624
H6A···H23C	2.6228	H23C···H11A^vi^	3.3821
H6B···H22A	3.5129	H23C···H21^vi^	3.2903
H6B···H22C	3.5968	H23C···H14^vi^	3.3666
H6B···H23A	2.3609	H24A···C11^x^	3.3429
H6B···H23B	3.4547	H24A···C22^xi^	3.1594
H6B···H23C	2.7893	H24A···C24^x^	3.5277
H9A···H24B	2.6196	H24A···H11A^x^	3.0146
H9A···H24C	3.0943	H24A···H11B^x^	2.7995
H9A···H25A	2.8127	H24A···H22A^xi^	3.2266
H9A···H25B	2.4175	H24A···H22B^xi^	2.6154
H9A···H25C	3.4909	H24A···H22C^xi^	3.1416
H9B···H11A	2.6782	H24A···H24A^x^	2.9515
H9B···H24A	3.4582	H24A···H24C^x^	3.3448
H9B···H24B	2.7656	H24B···C17^xi^	3.2834
H9B···H24C	2.3718	H24B···C22^xi^	3.4848
H9B···H25B	3.4917	H24B···C25^i^	3.4493
H11A···H24A	2.8548	H24B···H17^xi^	3.0741
H11A···H24B	3.4969	H24B···H22A^xi^	3.4712
H11A···H24C	2.4264	H24B···H22B^xi^	3.3381
H11A···H25A	3.5705	H24B···H22C^xi^	3.0828
H11A···H25C	3.5156	H24B···H25A^i^	2.9632
H11B···H24A	2.5422	H24B···H25C^i^	3.0634
H11B···H24B	3.5782	H24C···O2^i^	2.9582
H11B···H24C	3.0115	H24C···C12^i^	3.3615
H11B···H25A	2.8613	H24C···C25^i^	3.4838
H11B···H25B	3.5047	H24C···H11B^i^	3.2856
H11B···H25C	2.4387	H24C···H11B^x^	3.1883
H17···H18	2.3158	H24C···H24A^x^	3.3448
H17···H15	2.3633	H24C···H25A^i^	3.0052
H20···H21	2.3019	H24C···H25C^i^	3.1006
H21···H14	2.2169	H25A···C17^xii^	3.5780
H21···H15	3.5043	H25A···C18^xii^	3.1900
H22A···H23A	3.5125	H25A···C24^iii^	3.4227
H22A···H23B	2.9104	H25A···H9B^iii^	3.2393
H22A···H23C	2.4305	H25A···H17^xii^	3.2996
H22B···H23A	3.5530	H25A···H18^xii^	2.5705
H22B···H23B	2.4981	H25A···H24B^iii^	2.9632
H22B···H23C	2.9305	H25A···H24C^iii^	3.0052
H22C···H23B	3.5385	H25B···C15^xi^	3.3457
H22C···H23C	3.5014	H25B···C16^xi^	3.1228
H22C···H15	2.7633	H25B···C17^xi^	3.5459
H24A···H25A	3.5321	H25B···C18^xii^	3.5671
H24A···H25B	2.9335	H25B···C21^xi^	3.4071
H24A···H25C	2.4730	H25B···H17^xii^	3.2416
H24B···H25A	3.5373	H25B···H18^xii^	2.8446
H24B···H25B	2.4825	H25B···H22C^xi^	3.3101
H24B···H25C	2.9306	H25B···H15^xi^	3.5908
H24C···H25B	3.5402	H25C···C24^iii^	3.5105
H24C···H25C	3.5345	H25C···H22B^xi^	3.1132
H14···H15	2.62 (5)	H25C···H22C^xi^	3.3602
Cl1···H4A^vii^	2.9523	H25C···H24B^iii^	3.0634
Cl1···H6A^viii^	3.4267	H25C···H24C^iii^	3.1006
Cl1···H6B^vii^	3.0761	H14···C17^iii^	3.41 (3)
Cl1···H20^ix^	3.1167	H14···C23^ii^	3.31 (3)
O1···H1^i^	3.5308	H14···H17^iii^	3.0182
O2···H4B^ii^	3.0143	H14···H23A^ii^	2.9290
O2···H9B^iii^	3.0186	H14···H23B^ii^	2.8139
O2···H11A^iii^	3.3915	H14···H23C^iv^	3.3666
O2···H22B^ii^	3.2809	H15···O3^i^	2.82 (3)
O2···H23B^ii^	2.5279	H15···H1^i^	3.5668
O2···H24C^iii^	2.9582	H15···H25B^viii^	3.5908
O3···H6A^iii^	3.0391		
			
C7—O1—C8	118.15 (17)	C5—C4—H4A	108.827
C2—C1—C13	108.84 (18)	C5—C4—H4B	108.825
C2—C1—C14	111.3 (2)	H4A—C4—H4B	107.694
C13—C1—C14	109.5 (2)	C5—C6—H6A	108.843
C1—C2—C3	118.91 (19)	C5—C6—H6B	108.834
C1—C2—C7	122.5 (2)	C7—C6—H6A	108.840
C3—C2—C7	118.6 (2)	C7—C6—H6B	108.826
O3—C3—C2	121.0 (3)	H6A—C6—H6B	107.685
O3—C3—C4	121.3 (3)	C8—C9—H9A	108.954
C2—C3—C4	117.6 (2)	C8—C9—H9B	108.952
C3—C4—C5	113.7 (2)	C10—C9—H9A	108.954
C4—C5—C6	108.5 (2)	C10—C9—H9B	108.949
C4—C5—C22	107.8 (2)	H9A—C9—H9B	107.758
C4—C5—C23	111.8 (3)	C10—C11—H11A	108.624
C6—C5—C22	110.0 (2)	C10—C11—H11B	108.623
C6—C5—C23	110.1 (2)	C12—C11—H11A	108.627
C22—C5—C23	108.5 (3)	C12—C11—H11B	108.630
C5—C6—C7	113.65 (18)	H11A—C11—H11B	107.591
O1—C7—C2	122.4 (2)	C1—C14—H14	120 (2)
O1—C7—C6	111.26 (18)	C15—C14—H14	116 (2)
C2—C7—C6	126.3 (2)	C14—C15—H15	117 (2)
O1—C8—C9	110.9 (2)	C16—C15—H15	114 (2)
O1—C8—C13	122.8 (2)	C16—C17—H17	119.465
C9—C8—C13	126.3 (3)	C18—C17—H17	119.480
C8—C9—C10	113.1 (2)	C17—C18—H18	120.276
C9—C10—C11	108.5 (2)	C19—C18—H18	120.295
C9—C10—C24	109.5 (3)	C19—C20—H20	120.642
C9—C10—C25	110.3 (2)	C21—C20—H20	120.636
C11—C10—C24	110.2 (2)	C16—C21—H21	118.966
C11—C10—C25	109.3 (3)	C20—C21—H21	118.975
C24—C10—C25	109.0 (3)	C5—C22—H22A	109.468
C10—C11—C12	114.5 (2)	C5—C22—H22B	109.471
O2—C12—C11	121.7 (3)	C5—C22—H22C	109.474
O2—C12—C13	120.7 (3)	H22A—C22—H22B	109.480
C11—C12—C13	117.6 (3)	H22A—C22—H22C	109.469
C1—C13—C8	122.4 (3)	H22B—C22—H22C	109.466
C1—C13—C12	119.2 (2)	C5—C23—H23A	109.469
C8—C13—C12	118.3 (2)	C5—C23—H23B	109.479
C1—C14—C15	124.6 (3)	C5—C23—H23C	109.476
C14—C15—C16	128.1 (3)	H23A—C23—H23B	109.462
C15—C16—C17	119.2 (2)	H23A—C23—H23C	109.469
C15—C16—C21	123.3 (3)	H23B—C23—H23C	109.473
C17—C16—C21	117.5 (3)	C10—C24—H24A	109.468
C16—C17—C18	121.1 (3)	C10—C24—H24B	109.469
C17—C18—C19	119.4 (3)	C10—C24—H24C	109.475
Cl1—C19—C18	119.6 (3)	H24A—C24—H24B	109.469
Cl1—C19—C20	119.2 (2)	H24A—C24—H24C	109.479
C18—C19—C20	121.2 (3)	H24B—C24—H24C	109.467
C19—C20—C21	118.7 (3)	C10—C25—H25A	109.481
C16—C21—C20	122.1 (3)	C10—C25—H25B	109.472
C2—C1—H1	109.048	C10—C25—H25C	109.464
C13—C1—H1	109.049	H25A—C25—H25B	109.478
C14—C1—H1	109.033	H25A—C25—H25C	109.467
C3—C4—H4A	108.824	H25B—C25—H25C	109.466
C3—C4—H4B	108.827		
			
C7—O1—C8—C9	−172.00 (18)	O1—C8—C9—C10	160.40 (19)
C7—O1—C8—C13	9.1 (4)	O1—C8—C13—C1	3.6 (4)
C8—O1—C7—C2	−7.4 (4)	O1—C8—C13—C12	−179.6 (2)
C8—O1—C7—C6	171.84 (18)	C9—C8—C13—C1	−175.1 (3)
C2—C1—C13—C8	−15.6 (3)	C9—C8—C13—C12	1.6 (4)
C2—C1—C13—C12	167.64 (19)	C13—C8—C9—C10	−20.7 (4)
C13—C1—C2—C3	−162.82 (19)	C8—C9—C10—C11	44.0 (3)
C13—C1—C2—C7	17.3 (3)	C8—C9—C10—C24	164.34 (19)
C2—C1—C14—C15	41.8 (3)	C8—C9—C10—C25	−75.7 (3)
C14—C1—C2—C3	76.4 (3)	C9—C10—C11—C12	−52.8 (3)
C14—C1—C2—C7	−103.6 (3)	C24—C10—C11—C12	−172.7 (3)
C13—C1—C14—C15	−78.6 (3)	C25—C10—C11—C12	67.5 (3)
C14—C1—C13—C8	106.3 (3)	C10—C11—C12—O2	−145.5 (3)
C14—C1—C13—C12	−70.5 (3)	C10—C11—C12—C13	36.4 (4)
C1—C2—C3—O3	−2.9 (4)	O2—C12—C13—C1	−10.4 (4)
C1—C2—C3—C4	174.8 (2)	O2—C12—C13—C8	172.7 (3)
C1—C2—C7—O1	−6.9 (4)	C11—C12—C13—C1	167.7 (2)
C1—C2—C7—C6	174.0 (2)	C11—C12—C13—C8	−9.2 (4)
C3—C2—C7—O1	173.2 (2)	C1—C14—C15—C16	176.2 (2)
C3—C2—C7—C6	−5.9 (4)	C14—C15—C16—C17	−172.4 (3)
C7—C2—C3—O3	177.0 (3)	C14—C15—C16—C21	7.1 (4)
C7—C2—C3—C4	−5.2 (4)	C15—C16—C17—C18	177.9 (2)
O3—C3—C4—C5	−146.3 (3)	C15—C16—C21—C20	−178.6 (2)
C2—C3—C4—C5	36.0 (4)	C17—C16—C21—C20	0.8 (4)
C3—C4—C5—C6	−53.3 (3)	C21—C16—C17—C18	−1.6 (4)
C3—C4—C5—C22	65.9 (3)	C16—C17—C18—C19	0.9 (4)
C3—C4—C5—C23	−174.92 (19)	C17—C18—C19—Cl1	−179.8 (2)
C4—C5—C6—C7	42.2 (3)	C17—C18—C19—C20	0.6 (4)
C22—C5—C6—C7	−75.5 (3)	Cl1—C19—C20—C21	179.07 (16)
C23—C5—C6—C7	164.9 (3)	C18—C19—C20—C21	−1.3 (4)
C5—C6—C7—O1	166.44 (19)	C19—C20—C21—C16	0.6 (4)
C5—C6—C7—C2	−14.4 (4)		

Symmetry codes: (i) *x*−1, *y*, *z*; (ii) −*x*+5/2, *y*−1/2, −*z*+3/2; (iii) *x*+1, *y*, *z*; (iv) −*x*+3/2, *y*−1/2, −*z*+3/2; (v) −*x*+5/2, *y*+1/2, −*z*+3/2; (vi) −*x*+3/2, *y*+1/2, −*z*+3/2; (vii) *x*−1/2, −*y*+1/2, *z*+1/2; (viii) *x*+1/2, −*y*+1/2, *z*+1/2; (ix) −*x*+2, −*y*, −*z*+2; (x) −*x*+1, −*y*, −*z*+1; (xi) *x*−1/2, −*y*+1/2, *z*−1/2; (xii) *x*+1/2, −*y*+1/2, *z*−1/2.

### Hydrogen-bond geometry (Å, º)

**Table:**

*D*—H···*A*	*D*—H	H···*A*	*D*···*A*	*D*—H···*A*
C23—H23*B*···O2^v^	0.96	2.53	3.461 (4)	163

Symmetry code: (v) −*x*+5/2, *y*+1/2, −*z*+3/2.
